# Gene Flow Disruption and Population Declines in a Soil Arthropod in Fragmented Habitats

**DOI:** 10.1111/mec.17820

**Published:** 2025-06-11

**Authors:** Tammy Ai Tian Ho, Jeppe Bayer Pedersen, Anne Aagaard, Mads F. Schou, Jesper Bechsgaard, Derek Corcoran, Tove Hedegaard Jørgensen, Signe Normand, Trine Bilde

**Affiliations:** ^1^ Section for Genetics, Ecology & Evolution, Centre for Ecological Genetics, Department of Biology Aarhus University Aarhus Denmark; ^2^ Section for Ecoinformatics and Biodiversity, Center for Sustainable Landscapes Under Global Change, Department of Biology Aarhus University Aarhus Denmark; ^3^ Centre for Ecology & Conservation University of Exeter Exeter UK

**Keywords:** arthropoda, conservation genomics, ecological genetics, gene flow, landscape fragmentation, population genomics

## Abstract

The intensification of land use over past millennia has accelerated habitat loss and fragmentation. This is hypothesized to lead to reductions in population sizes and restrictions in gene flow, processes that amplify genetic drift with profound negative impacts on species and populations. However, empirical data on the population genetic impacts of habitat fragmentation remain limited, particularly for presumed abundant species such as insects. Reports of dramatic insect and arthropod declines are increasing, and their short generation times and limited dispersal capacities make them especially vulnerable to habitat fragmentation. To substantiate the hypothesis that habitat fragmentation negatively impacts genetic composition and demography, we combined historical agricultural land use data from Denmark with whole‐genome resequencing of 25 populations of the collembolan *Entomobrya nicoleti* from natural grasslands. Abundance data indicate that agricultural expansion reduces habitat suitability and fragments populations. Demographic modelling shows that intensification of agricultural land use coincides with severe declines in effective population sizes. It is likely that these declines have yet to reach their full effect on current levels of genetic diversity because of the ‘drift debt,’ where the genetic diversity of recently declined populations will erode over future generations. Gene flow estimates revealed sharp recent declines that coincide with agricultural intensification. Our results underscore that even seemingly abundant species in fragmented landscapes can experience severe reductions in effective population size and gene flow. These demographic shifts predict future genetic erosion, highlighting the delayed yet inevitable consequences of habitat fragmentation for population persistence.

## 1 Introduction

Understanding how environmental change influences genetic diversity within and among populations is of fundamental importance in conservation genetics. The demographic history of species determines their genetic composition and is shaped by past climatic events such as ice ages and more recent anthropogenic impacts such as land use change and pollution (Banks et al. 2013; Ellegren and Galtier 2016; *IPBES* 2019). Intensive land use has caused widespread habitat loss and fragmentation, which predicts profound negative impacts on the sizes and connectivity of populations (Cushman 2006; Donald et al. 2006; Jaureguiberry et al. 2022; Riva et al. 2024; Seibold et al. 2019; Turner 2010). Genetic diversity may decrease as a consequence, and this will ultimately threaten the adaptive potential and persistence of populations (Charlesworth et al. 2017; Frankham 2005; Ørsted et al. 2019; Willi et al. 2006). Many natural habitats in Europe have been gradually reduced and fragmented over the past several millennia due to the cultivation of landscapes, and the accelerating intensification of agriculture during the past few hundred years has exacerbated these effects (Goldewijk et al. 2011). However, we have limited knowledge of the consequences of these land use changes for the genetic composition within and between populations (Banks et al. 2013; Ellegren and Galtier 2016).

Loss of habitat directly causes reductions in effective population sizes (N_e_), which is associated with enhanced genetic drift and stochastic loss of genetic variants (Lynch et al. 1995). This effect is exacerbated by habitat fragmentation, as unsuitable habitat between the fragments can act as barriers to gene flow by impeding the movement of individuals between populations (Stevens et al. 2018). If populations remain small and isolated, they lose adaptive potential and are furthermore expected to show increased expression of genetic load (inbreeding depression) (Bertorelle et al. 2022; Kardos et al. 2021). The degree to which populations are impacted by these processes depends on demographic and biological properties of the species. The current level of genetic diversity of a species is strongly influenced by its demographic history (Ellegren and Galtier 2016; England et al. 2003; Garner et al. 2005; Gustafson et al. 2017), and both past and more recent population declines will be reflected in current population genetic composition. Life history traits such as generation time, longevity, and reproductive strategy also contribute to the genetic diversity of species so that relatively short‐lived or highly fecund species are more diverse than long‐lived or low‐fecund species (Hartfield 2016; Romiguier et al. 2014; Slotte et al. 2013; Welch and Meselson 2000). Dispersal capacity is a trait that can potentially buffer against habitat fragmentation so that species with high dispersal capacity are expected to be better able to maintain gene flow among seemingly isolated populations (Cushman et al. 2015). This implies that species with different life history strategies may respond differently to environmental disturbances, which should be reflected in the current levels of population genetic diversity and its structure.

Much conservation genetic research focuses on endangered species that exist in small populations subject to genetic drift and loss of genetic diversity (Charlesworth and Charlesworth 1987; Kardos et al. 2021; Lynch et al. 1995), while population genetic studies of abundant and widespread species are fewer. Arthropods are often presumed to exist in large populations and therefore potentially less negatively impacted by habitat loss and fragmentation. On the other hand, arthropods may be vulnerable to environmental perturbations due to their small sizes and short generation times. In the last decades, reports on massive declines in insects and other arthropods have accumulated (e.g., Wagner 2020; Webster et al. 2023), including reports on disproportionally high losses of common and abundant insect species (van Klink et al. 2024). However, we have almost no data on whether and how these declines impact on population genetic diversity and structure (e.g., Hoffmann et al. 2021). Such information is crucial for evaluating the fitness effects of segregating variants and thereby the ‘genetic health’ of populations. This is concerning not only for biodiversity loss, but also for ecosystem function as arthropods are vital for trophic interactions and for a broad range of functions such as decomposition, pollination, biological control etc. (Kremen 2018; Losey and Vaughan 2006; Vanbergen et al. 2013; Wagner 2020; Yang and Gratton 2014).

Collembola (springtails) is an important group of soil‐dwelling arthropods with global distribution, comprising up to 32% of the abundance of all terrestrial arthropods (Potapov et al. 2024, 2023; Rosenberg et al. 2023). Springtails have important ecological functions in the decomposition of organic matter (Bardgett et al. 1993; Seastedt 1984). Although they often exist in large population sizes, springtails are flightless, have limited movement capacity, are sensitive to desiccation, and are therefore expected to be poor active dispersers (Costa et al. 2013; Hopkin 1997). This results in limited gene flow and genetic structure on larger intra‐continental scales (Collins et al. 2020; Saltzwedel et al. 2016; Timmermans et al. 2005), and to some extent also on local scales (tens of kilometres) (Cicconardi et al. 2010; Garrick et al. 2004; Roberts and Weeks 2011; Wurff et al. 2005). Our study species, *Entomobrya nicoleti*, has a global distribution (Bellinger et al. 2024a, 2024b). Collembolans and soil arthropods are sensitive to agricultural practices such as tillage regimes (Frampton 2000) and may suffer high mortality from mechanical soil treatment (Axelsen et al. 2022; Thorbek and Bilde 2004). *E. nicoleti* is found in open terrestrial habitats across Denmark (this study) and is therefore a suitable model system for investigating hypothesised effects of land use and habitat fragmentation on demography and genetic structure.

**FIGURE 1:**
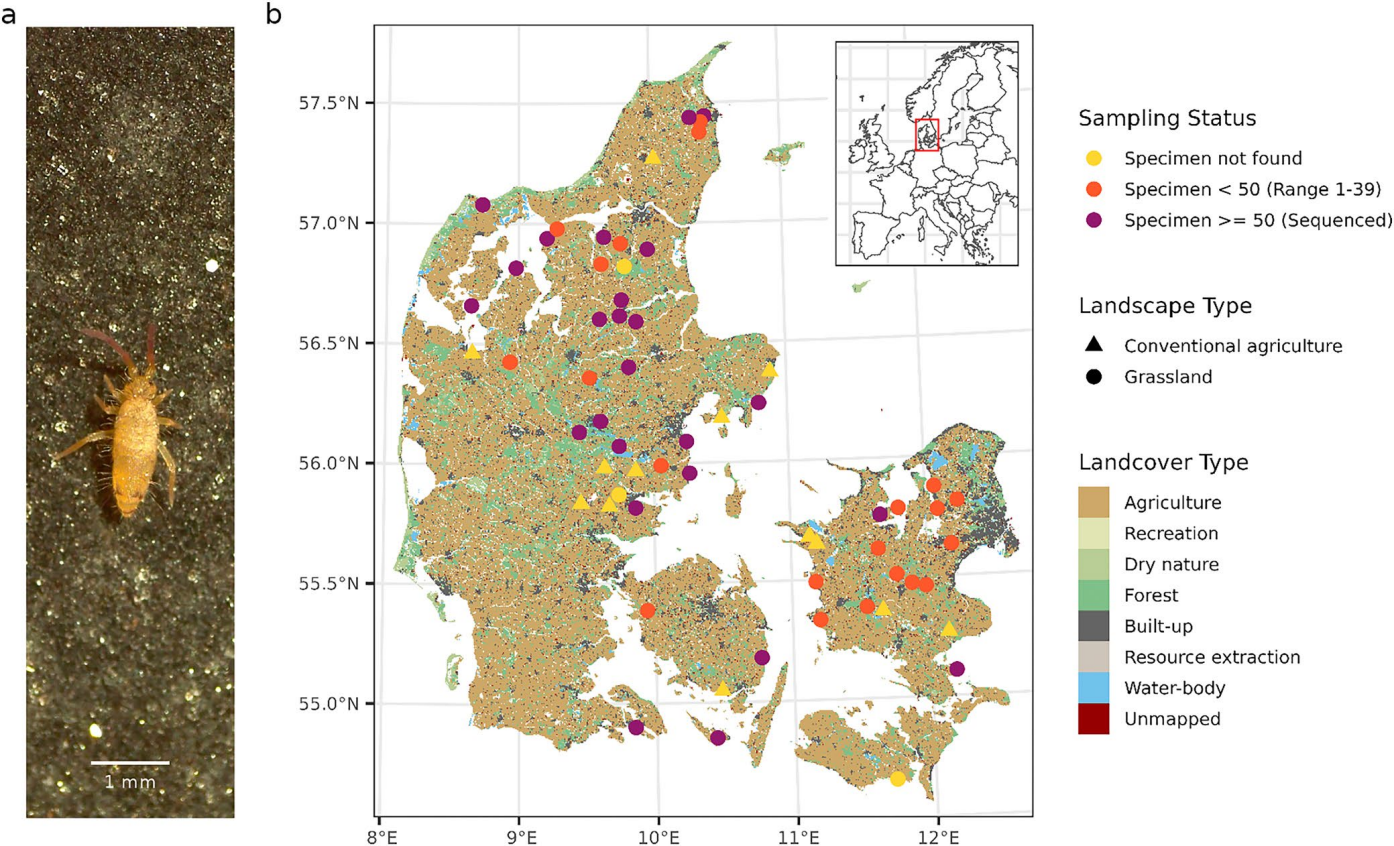
Study species and sampling sites across Denmark. We sampled *Entomobrya nicoleti* (a) across 49 non‐acidic grasslands, that is, grasslands on more or less calcareous soils (Staalsen 2024), in Denmark (b) with the aim of obtaining 50 specimens from each site. The purple dots on the map (b) represent sites where this goal was reached, and the specimens sequenced and analysed (N=25), while orange dots represent sites where less than 50 individuals were found (N=21). Yellow dots/triangles represent grasslands where no specimens were found (N=3). We furthermore sampled 13 conventional agricultural fields (triangles) to evaluate if they represent barriers to *E. nicoleti*, and did not find any specimens in any of those fields (Table S1).

Denmark is one of the most intensively cultivated land areas in the world (Lohrum et al. 2024; Nielsen et al. 2021). Small‐scale cultivation started approximately 6000 years ago, while the intensification of agricultural production substantially changed the landscape during the last two centuries due to the conversion of natural grasslands to fields and intensive drainage of wet meadows (Lohrum et al. 2024; Nielsen et al. 2021). These landscape transformations substantially increased the fragmentation of natural habitats as well as reducing their quality due to the increased use of nutrients, pesticides, and tillage. The resulting fragmentation and habitat disturbance are expected to have impacted populations of soil arthropods. In the present study, we sampled populations of the collembola *E. nicoleti* in 49 grasslands and 13 conventional agricultural fields located across Denmark (Figure 1). We generated *de novo* assembly of the *E. nicoleti* genome and performed whole‐genome pooled resequencing of 50 individuals from each of 25 grassland populations where a sufficient number of individuals were obtained. Finally, we generated a comprehensive dataset of temporal land use changes in Denmark to provide historical context for interpreting population genetic patterns associated with land use change. We used these comprehensive datasets combined with genetic and demographic modelling to assess and substantiate the hypothesis that the intensification of agricultural land use in historical time is associated with changes in population genetic parameters, effective population sizes and rates of gene flow.

## 2 Methods

### 2.1 Sampling

We sampled 55 non‐acidic grasslands, that is, grasslands on more or less calcareous soils (Staalsen 2024), (49 analysed, see below) and 13 conventional agricultural fields (Figure 1) between April 2022 and October 2023 using an inverted leaf blower (*STIHL SH86 C‐E blower; Stihl, Waiblingen, Germany*). A custom‐made sock made of thin cotton was inserted in the vacuuming tube to collect all animals including small collembolans. The vacuum sampler was pulsed to the ground approximately 300 times at random locations at each site on each sampling occasion. Grasslands were sampled 5–6 times, conventional agricultural fields were sampled one or two times each (1.85 on average) during the 7‐month sampling period (Table S1). The socks were emptied into plastic boxes (16 × 16 × 11 cm) immediately after sampling, stored temporarily in a cooling box (approximately 5°C for maximum 3 days) or brought directly back to laboratory storage (Aarhus University, Denmark) at 4°C for further processing. Within 5 days of sampling, all live collembolans were collected in petri dishes on moist plaster of paris, then frozen at −18°C and sent to taxonomists for species identification. To verify that our sampling method is representative for a given location, we sampled 50 individuals on two separate sampling trips from one site (SKJ), did pooled sequencing on both samples, and independently generated estimates for both. The demographic modelling estimates from these two sampling occasions were highly similar (see Figure S1) verifying our method, and we subsequently used only one of these samples in our analyses.

### 2.2 DNA Extraction and Sequencing

#### 2.2.1 DNA for Reference Genome Sequencing

DNA from 50 pooled individuals was extracted using the MagAttract HMW DNA kit from Qiagen (Hilden, Germany). The 50 individuals used for genome sequencing came from a population at Aarhus University campus; this population was not included in the analysis. Initially, we extracted DNA from 1 and 10 individuals, but did not obtain enough DNA for PacBio sequencing. We therefore extracted from 50 individuals to obtain sufficient DNA. Samples were initially homogenised in lysis buffer (ATL buffer) using a pellet pestle, proteinase K was added, and the sample was incubated at 56°C for 3 h. The rest of the extraction followed the manufacturer's protocol. The DNA was sequenced using PacBio sequencing to 25.5 Gb HiFi read data.

#### 2.2.2 DNA for Re‐Sequencing

DNA from 50 individuals of similar size (evaluated by eye) from each field was extracted in pools using the DNeasy Blood and Tissue kit from Qiagen. Samples were initially homogenised in lysis buffer (ATL buffer) using a pellet pestle, proteinase K was added, and the sample was incubated at 56°C for 3 h. The rest of the extraction followed the manufacturer's protocol. Each DNA extract was sequenced using DNBSEQ PE150 (BGI) to obtain at least 150 Gb raw data. It is a concern that unequal contribution of DNA to the pool can be a problem when performing pool‐seq. The inclusion of a large number of individuals in each pool, here 50 individuals, should minimise any effect from an unequal contribution of DNA (Gautier et al. 2013).

#### 2.2.3 DNA for Hi‐C Sequencing

We used the Dovetail Omni‐C kit provided by Catania Bio to generate Hi‐C data for contig scaffolding. The Omni‐C library was sequenced using DNBSEQ PE150 (BGI). About 50 individuals were used.

### 2.3 Data Analyses

#### 2.3.1 Reference Genome Assembly and Annotation

Before the assembly process, any potential remaining adapters were removed from the PacBio HiFi sequencing data using HiFiAdapterFilt (Sim et al. 2022). A draft genome assembly was carried out using hifiasm (Cheng et al. 2021, 2022) with a similarity threshold of 0.1 and an initial purge level of 3 (‐s 0.1 ‐l 3 –primary). Further haplotypic duplication and overlaps were identified using purge_dups (three rounds) (Guan et al. 2020) with minimap2 (Li 2018) and the resulting draft genome was assessed with BUSCO (Manni et al. 2021) using arthropoda_odb10. Contigs were scaffolded with Hi‐C data using the Juicer pipeline (Durand, Shamim, et al. 2016) (to create a Hi‐C contact map) followed by the 3D‐DNA pipeline (Dudchenko et al. 2017) (to correct mis‐assemblies, anchor, order and orient fragments of DNA). The resulting assembly was then reviewed and manually curated using Juicebox Assembly Tools (Durand, Robinson, et al. 2016) and then reassessed with BUSCO. Two separate assemblies were made; one consisting only of chromosome‐level scaffolds, and one containing all contigs. At chromosome level, it was possible to scaffold 3187 contigs into 7 chromosomes, comprising a total size of 254.1 Mb, achieving a BUSCO completeness score of 50.9%. However, a further 3273 contigs, comprising 115.2 Mb, remained of the data, which were not possible to scaffold satisfactorily, but when included, increased the BUSCO completeness score to 93.9%. It was decided that all further analyses would be undertaken only on the parts of the genome that could be successfully scaffolded. We consider the scaffolded genome to be representative and sufficient for the aims of this study. A table of summary statistics for both the final genome assemblies is available in the Supporting Information (Table S2).

Repetitive content in the genome was identified using RepeatMasker (Smit et al. 2013) combining the results from two separate runs, with the results from RepeatModeler (Flynn et al. 2020) and an arthropod dataset from RepBase (Bao et al. 2015) respectively, and soft‐masked using bedtools (Quinlan and Hall 2010).

The protein coding genes were annotated using BRAKER (Brůna et al. 2021, 2020; Buchfink et al. 2015; Gabriel et al. 2021; Gotoh 2008; Hoff et al. 2016, 2019; Iwata and Gotoh 2012; Lomsadze et al. 2005; Stanke et al. 2008, 2006). The annotation was based on protein coding sequences from another collembolan, *Orchesella cincta* (Entomobryidae). These data were downloaded from NCBI (GCA 001718145.1^2016^) and the protein‐coding sequences were extracted using AGAT (Dainat 2023).

#### 2.3.2 Mapping, Filtering and Variant Calling of Resequencing Data

Prior to mapping, the re‐sequencing data were processed with AdapterRemoval (Schubert et al. 2016) to search for and remove any residuals of known adapter sequences. Furthermore, reads were trimmed at the 5′ and 3′ termini for Ns and bases with a quality score lower than 25, reads shorter than 20 base‐pairs were also discarded, and paired‐end reads overlapping mates were merged into a single read and the base quality was recalculated if the overlap was at least 11 base pairs in length (default) with a maximum mismatch ratio of $\frac{1}{3}$ (default). The resequencing data was mapped to the reference genome using the BWA‐MEM algorithm from BWA (Li 2013). Duplicates were marked, and alignment files filtered using samtools (Danecek et al. 2021). During filtering all unmapped reads, non‐primary reads, failed reads, duplicates and supplementary reads were removed (excluded bit‐flag value: 3844). Assessment of alignment data quality post‐filtering was done using Qualimap (García‐Alcalde et al. 2012).

Variants were called for each of the populations using freebayes (Garrison and Marth 2012) with the parameters: ‐n 3 ‐p 100 –min‐alternate‐fraction 0 –min‐alternate‐count 2 –pooled‐discrete. VCF files were then filtered using BCFtools (Danecek et al. 2021) according to the following: (1) removing 5 bp on each side of indel polymorphisms as the uncertainty of alignments, and consequently variant calls, are increased close to indels, (2) removing all indels as we are interested in SNPs, (3) removing sites with coverage lower than 301 to ensure the ability to call even rare variants and higher than 599 to avoid mtDNA and maintain comparability, (4) removing sites that were not bi‐allelic as true multi‐allelic sites are expected to be rare. For all subsequent analyses, we used genomic regions not including protein coding genes (exons and introns) and repetitive regions. This was done to analyse regions as neutrally as possible to avoid patterns generated by selection.

#### 2.3.3 Estimation of Population Differentiation (F_st_)

To estimate neutral divergence across the genome, F_st_ for each population pair was estimated on allele frequencies using the equation employed by the PoPoolation script fst_sliding.pl. (see script help section, Kofler et al. 2011). Only intergenic, non‐repetitive, bi‐allelic sites were included in the F_st_ estimation (see filtering above). For assessing isolation‐by‐distance (IBD), the geographical distance between the populations were calculated using R v. 4.3.3 (R Core Team 2024) and the function distm() from the Geosphere package v.1.5‐18 (Hijmans, Karney, et al. 2024). A UPGMA cladogram was built based on F_st_ values using the hclust() function with method ‘average’ from the R stats package and plotted using plot() and the function set() from the dendextend v.1.17.1 package (Galili 2015).

#### 2.3.4 Estimation of Nucleotide Diversity

Population level nucleotide diversity (π) was estimated on intergenic and non‐repetitive sites, including non‐variable and bi‐allelic sites (see variant filtering above). Using allele frequencies to estimate π, we used Nei's π equation with sample size correction ($\pi =2\times \text{freq}\left(A\right)\times \text{freq}\left(a\right)\times \frac{N}{N-1}$) (Nei and Tajima 1981), where $\text{freq}\left(A\right)$ and $\text{freq}\left(a\right)$ are the frequency each of the alternative alleles and N is the number of chromosomes.

#### 2.3.5 conStruct Analysis

We estimated admixture among populations using the R‐package conStruct v. 1.0.6 (Bradburd 2024). conStruct detects genetic covariance across samples that are in accordance with continuous isolation‐by‐distance (IBD), and then identifies discrete layers of covariance that contribute to genetic clustering of populations beyond IBD. For this analysis, we used allele frequencies of all polymorphic positions in the genome that were kept after filtering the VCF files. Only sites found across all populations were kept (no missing data). We evaluated models with and without IBD, and with 1–5 spatial layers (K's) using cross‐validation in 16 replicate models. The chain mixing across the different replicates was observed to be satisfactory when using 400,000 iterations, which also resulted in high convergence across replicates. The best model was determined by comparing predicting accuracy and inspecting the layer contributions (Figure S2).

#### 2.3.6 Demographic History

To estimate the recent demographic history of each population we used Stairway Plot v2.1 (Liu and Fu 2020). Folded site frequency spectra (SFSs) were calculated from the VCF file of each population (Figure S3, scripts pertaining to the calculation of folded SFSs are available at https://github.com/EcologicalGenetics/gene_flow_disruption_and_population_fragmentation_in_a_soil_arthropod_in_fragmented_habitats). None of the SFS show signatures of population structure or recent bottleneck (Barroso et al. 2020). Since no mutation rate estimate has been estimated for any collembola species, we assumed a mutation rate of $2.5\times 10^{-9}$ based on estimates from insect species (Keightley et al. 2015; Liu et al. 2016). It is undocumented what the generation time of *Entomobrya nicoleti* is, however, many collembola species have a generation time of about 1 month in the lab, among them *Folsomia candida* , which is similar in size to *E. nicoleti*. It is well known that processes speed up under laboratory conditions causing somewhat shorter generation times. For example, in *Drosophila melanogaster* that has about 10 generations per year under natural conditions (Christiansen et al. 1992), generation time is about 13 days under laboratory conditions at 20°C. Based on this we assume a generation time of 4 months under natural conditions for *E. nicoleti*. All Stairway Plot analyses were run excluding singletons as recommended by the authors of the program (Liu and Fu 2020). The estimated demographic history was z‐transformed using the R function scale() and averaged, to encapsulate the common demographic trajectory of all populations. To calculate a confidence interval along the curve, we bootstrapped on the level of populations 10,000 times, and calculated the average across z‐transformed population medians. The confidence interval was calculated as the 2.5% and 97.5% quantiles of the bootstrapped data.

**FIGURE 2:**
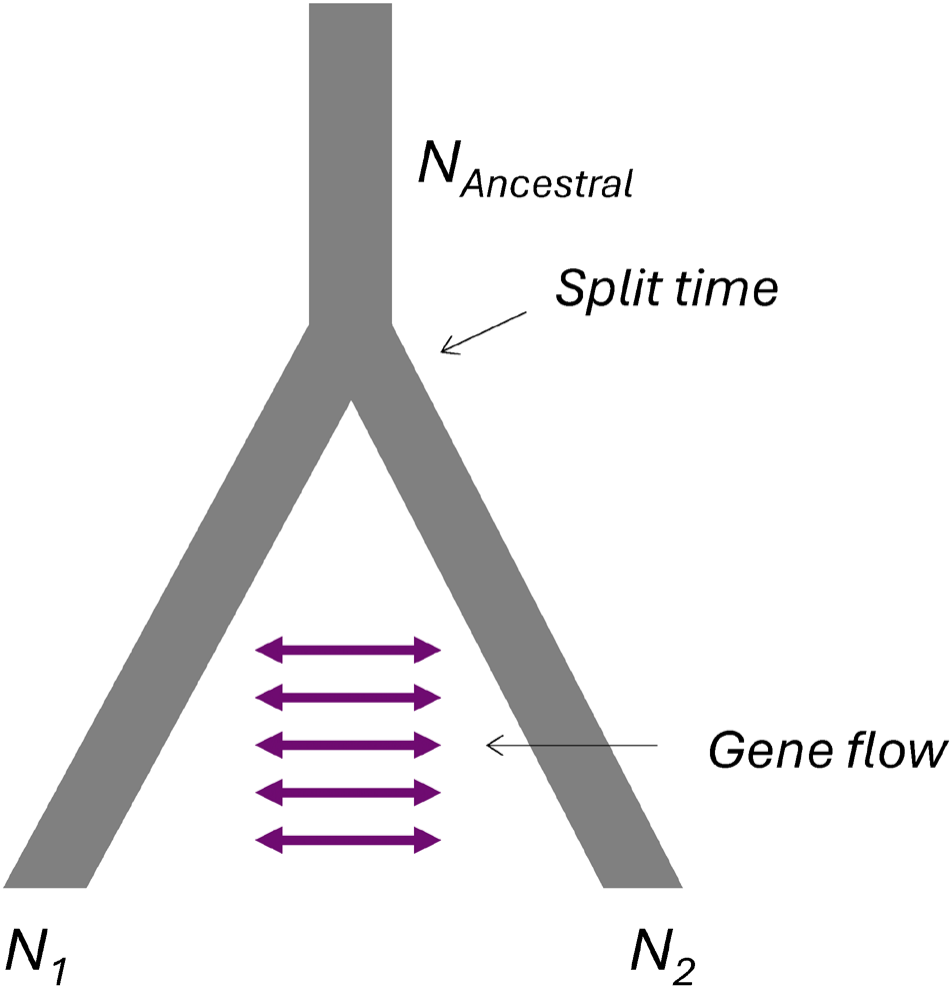
Illustration of demographic scenario. Figure illustrating the demographic scenario and parameters estimated by fastsimcoal2. N1 and N2 are current effective population sizes of two populations after a split. NAncestral is the ancestral effective population size prior to the split. Split time indicates the time before present when the two current population merged into one ancestral population. Gene flow, as indicated by the purple double‐sided arrows, shows potential gene flow between the populations at intervals between present and when the split occurred.

#### 2.3.7 Gene Flow

We estimated the rates of gene flow among populations (average between all pairs of populations) as a function of time intervals of 100 generations over the last 14,000 generations. This corresponds to 4666 years assuming 3 generations per year. This period covers both the start and the intensification of agriculture in Denmark. To estimate gene flow over time, we first estimated joint site frequency spectra (2dSFS) for all pairs of populations using a custom script (see https://github.com/EcologicalGenetics/gene_flow_disruption_and_population_fragmentation_in_a_soil_arthropod_in_fragmented_habitats) operating on the filtered VCF file (see above), and used fastsimcoal2 (Excoffier et al. 2013) to estimate a set of demographic parameters for each pair of populations in an isolation with migration model (Figure 2). We included two gene flow rate parameters during the period between present and the time when the two current populations merged into the ancestral population; one gene flow rate estimated during the period from present to X generations ago (t_2_ in Figure S4f), and another gene flow rate estimated during the period from X generations ago to when the two populations merge into an ancestral population (t_1_ in Figure S4f). X was varied between 100 and 14,000 generations in intervals of 100 generations (I).

The full set of parameters estimated with fastsimcoal2 were: effective population sizes of each of the two populations, the ancestral population size (all population sizes were assumed to be constant), the time to the merging of the two current populations into an ancestral population, and the two rates of gene flow described above (Figure 2 and Figure S4f). Gene flow was assumed to be symmetrical. The prior distributions of the demographic parameters were: current and ancestral population sizes: 10–10,000,000, divergence time: 10–100,000 generations, and gene flow rates: probability of a gene moving from one population to the other of $1\times 10^{-20}$ to $1\times 10^{-1}$ per generation. The mutation rate was assumed to be $2.5\times 10^{-9}$ (based on Liu et al. 2016; Mackintosh et al. 2019). We ran all fastsimcoal2 analyses with at least 100,000 simulations (‐n 100000), maximum likelihood estimation (‐M) with 40 ECM cycles (‐L 40) of minor allele SFS (‐m). Analyses were conducted both with and without singletons, to test if the potential uncertainties in calling singletons affected our gene flow rate estimates (see Figure S6). We found that excluding singletons shifted the timing of change in gene flow rate slightly back in time, but this does not affect the main conclusion. We present the results that include singletons. If the estimate of the time that the two populations merge into the ancestral population was lower than the for each run preset X (number of generations before present), the analyses were removed (∼ 15%, 6135 of 40,210 estimates), to avoid including gene flow estimates in the ancestral population.

We converted the gene flow rate estimates integrated over the recent period (t_2_ in Figure S4f) into estimates of gene flow rates at each time interval (I) of 100 generations by fitting a smoothed line to t_2_ gene flow estimates (averaged across estimates of all population pairs) using the R base function smooth.spline(). Since our gene flow estimates are integrated over n intervals (I), from present to X, we assume that the fitted value equals the average of the actual values, from $I_{1}$ ($\mathrm{Act}\left(I_{1}\right)$) to $I_{n}$ ($\mathrm{Act}\left(I_{n}\right)$):

(1) $$\mathrm{Fit}\left(X\right)=\frac{\mathrm{Act}\left(I_{1}\right)+\mathrm{Act}\left(I_{2}\right)+\cdots +\mathrm{Act}\left(I_{n}\right)}{n}$$

for each I, we want to know the actual value:

(2) $$\mathrm{Act}\left(I_{n}\right)=\mathrm{Fit}\left(X\right)\times n-\sum \mathrm{Act}\left(I_{1}\right)+\mathrm{Act}\left(I_{2}\right)+\cdots +\mathrm{Act}\left(I_{n-1}\right)$$

Using this principle, we calculated gene flow rates for each time interval (I) of 100 generations from the fitted line representing our fastsimcoal2 t_2_ gene flow estimates in an iterative manner starting with X=100, followed by X=200, etc. Example of the calculations: The rationale of the calculations done in Formula (1) and (2) is that each estimate of t_2_ is nested in the next estimate(s) of t_2_. For example, $t_{2\left(\text{present}\to X_{500}\right)}$ is nested in $t_{2\left(\text{present}\to X_{600}\right)}$. We can therefore calculate what the rate of gene flow in each interval (of 100 generations) must have been to change the average between two consecutive *t*_2_ estimates. If the $t_{2\left(\text{present}\to X_{500}\right)}$ (which is estimated across five intervals of 100 generations) was estimated to be 0.003 and $t_{2\left(\text{present}\to X_{600}\right)}$ (which is estimated across six intervals of 100 generations) to be 0.0035, the gene flow rate in the time interval between generation 500 and 600 must have been:

$$\begin{array}{c} \frac{5\times 0.003+{\text{geneflow}}_{500\to 600}}{6}=0.0035\\ ⇓\\ {\text{geneflow}}_{500\to 600}=0.006 \end{array}$$

To calculate a confidence interval, we bootstrapped our population pair gene flow estimate trajectories and repeated the analysis above on the bootstrapped set of population pairs. This was repeated 10,000 times, and the resulting confidence interval was calculated as the 2.5% and 97.5% quantiles.

To verify that gene flow can be meaningfully estimated by utilising fastsimcoal2 as described above, we simulated neutral 2dSFS (50 diploid individuals per population) of five demographic scenarios using fastsimcoal2 (Excoffier et al. 2013). When running the same fastsimcoal2 analysis to estimate demographic parameters based on simulated 2dSFSs as we did on observed 2dSFSs, we can evaluate how well this analysis can estimate the input parameters used to simulate 2dSFS in each scenario. All five scenarios included two populations both with a current size of 22,500 (11,250 diploid) that split up 45,000 generations ago, an ancestral population size of 405,000 (202,500 diploid), and with six chromosomes of 15,000,000 bases each, a recombination rate of $1\times 10^{-8}$, and a mutation rate of $1\times 10^{-8}$. *Scenario 1* was simulated without gene flow. *Scenario 2* was simulated with constant symmetrical gene flow of $6\times 10^{-5}$ (probability that a gene moved from one population to the other each generation). *Scenario 3* was simulated with constant symmetrical gene flow of $6\times 10^{-5}$ and exponentially declining population size with a rate of 0.00005 per generation (totalling an approximate 90% reduction) since the split of the two populations (current size being 1000 diploid individuals). *Scenario 4* was simulated with constant symmetrical gene flow of $6\times 10^{-5}$ and exponentially increasing population size with a rate of 0.00005 per generation (totalling an approximate 10‐fold increase) since the split of the two populations (current size being 500,000 diploid individuals). *Scenario 5* was simulated with decreasing symmetrical gene flow over the last 12,000 generations (gene flow of $6\times 10^{-5}$ from population split until 12,000 generations ago, of $5\times 10^{-5}$ from 12,000 to 9000 generations ago, of $4\times 10^{-5}$ from 9000 to 6000 generations ago, of $3\times 10^{-5}$ from 6000 to 3000 generations ago, of $2\times 10^{-5}$ from 3000 to 600 generations ago, of $1\times 10^{-5}$ and of 0 between 600 generations ago until present). Each scenario was simulated with 100 replicates. See Figure S7 for a visualisation of the simulated scenarios. The parameter files used as input in the fastsimcoal2 simulations are available at: https://github.com/EcologicalGenetics/gene_flow_disruption_and_population_fragmentation_in_a_soil_arthropod_in_fragmented_habitats.

#### 2.3.8 Land Use Development During the Last 4000 Years in Denmark

The vegetation in the majority of Denmark developed from tundra to grasslands and forests as the climate warmed after the ice sheet melted 11,700 years ago (Odgaard 2015). The onset of agricultural practices occurred approximately 6000 years ago.

To investigate historical land use changes in Denmark, we utilised the HYDE 3.3 baseline dataset (Goldewijk et al. 2017), accessed through the pastclim R package (Leonardi et al. 2023). This dataset provides global reconstructions of regional land use patterns, including cropland, grazing land, pasture, and urban areas, at a spatial resolution of 5 arc minutes and a temporal resolution spanning thousands of years. It is important to note that the dataset provides estimates of land use cover at the scale of landscapes and regions but does not capture small‐scale changes in land cover or the onset of agriculture. Hence, the estimates of change in agricultural area are expected to represent changes in fragmentation and be of relevance for understanding the population genetic structure. For this study, we summed the total area of regional land use change at the national scale.

Spatial data for Denmark were extracted using administrative boundaries from the Global Administrative Areas Dataset from GADM v4.1 (GADM, 2024) accessed via the *geodata* R package (Hijmans 2023) and were constrained to Denmark's extent by cropping and masking rasters with the terra package (Hijmans, Bivand, et al. 2024). For each time step, we calculated the total area (in square kilometres) of each land use type and collated these into a comprehensive dataset to assess temporal land use transitions. These analyses were conducted in R (R Core Team 2024) and provided critical historical context for interpreting population genetic patterns associated with habitat fragmentation.

## 3 Results

### 3.1 Specimen Sampling

We sampled *Entomobrya nicoleti* (Figure 1a) in both grasslands and conventional agricultural fields across Denmark. The species was found in most grasslands but not in conventional agricultural fields (Figure 1b, Table S1). Since agricultural fields cover about 60% (light brown area on the map) of Denmark's area, this indicates that the landscape is highly fragmented and that conventional agricultural fields could effectively prevent or reduce gene flow of *E. nicoleti*.

### 3.2 Land Use Development Over Time

**FIGURE 3:**
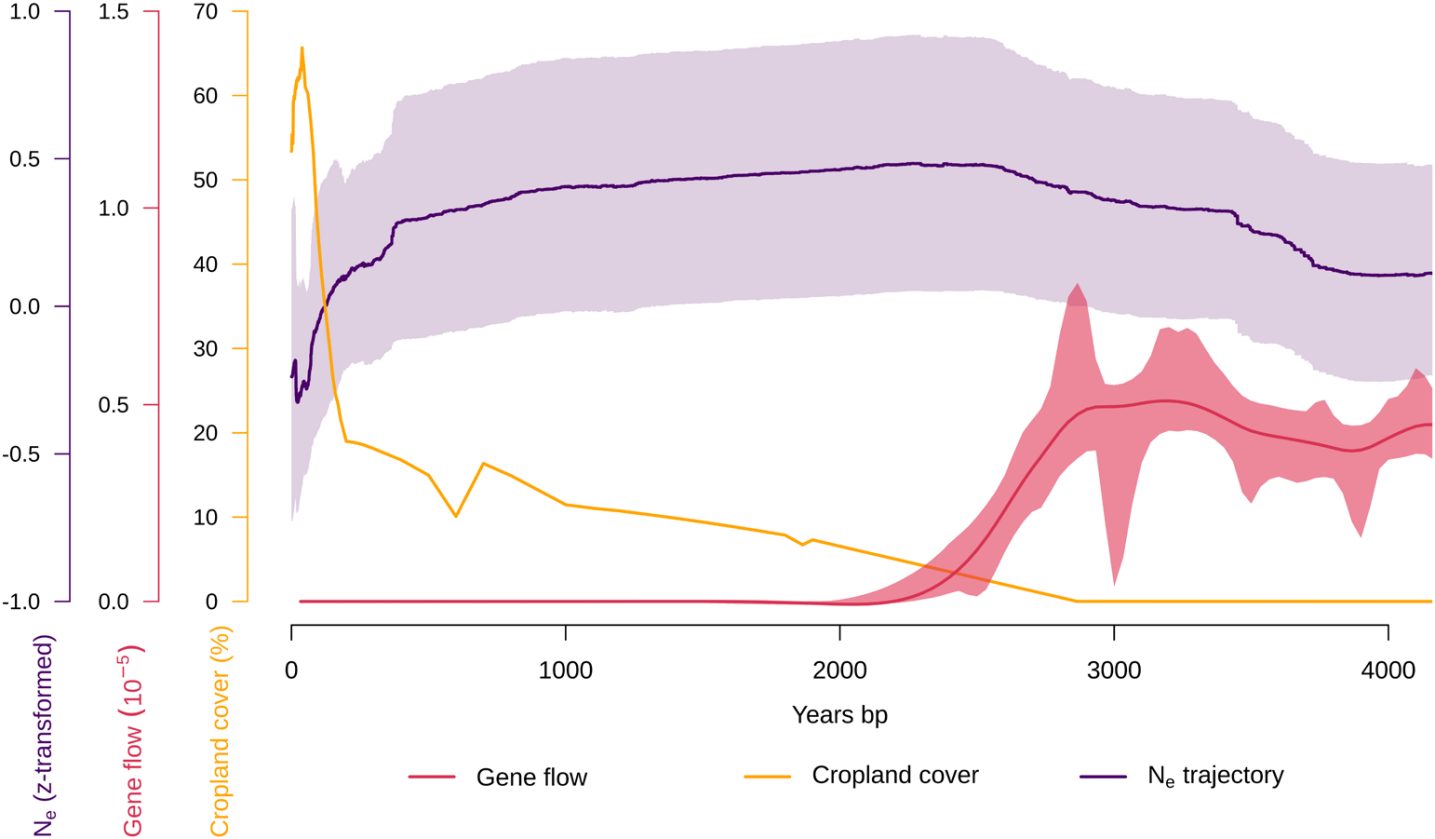
Changes in gene flow, cropland cover, and population demography over the last 4000 years. The figure shows trajectories of (1) effective population size for all populations represented as a mean of z‐transformed trajectories including 95% CI (purple), (2) agricultural history, presented as percentage of cropland cover (orange), (3) predicted gene flow rate including 95% CI (red), based on estimates from the fastsimcoal2 analysis described in the methods 2.3.7.

Our analysis of historical land use changes in Denmark, using the HYDE 3.3 baseline dataset (Leonardi et al. 2023), reveals large changes in the cover of cropland over the last 4000 years. For approximately 3000 years ago regional change in cropland started to increase (Figure 3). The increase continued until ∼ 200 years ago where intensification greatly accelerated (Figure 3).

By the start of the industrial era (∼ 200 years ago), cropland reached its peak, accounting for the majority of converted natural land, with approximately 28,289 km^2^ transformed over time. Grazing land and pasture followed, with 3551 and 3394 km^2^ converted, respectively. Urban areas, while initially limited, grew exponentially during the last millennium, adding 1357 km^2^ in total and accelerating significantly in the last 200 years. On average, cropland accounted for nearly 60% of converted land, highlighting its dominance in shaping Denmark's landscapes.

These changes in agricultural areas align closely with historical records of land use intensification and likely contributed to increased habitat fragmentation. This fragmentation is particularly evident in recent centuries, where rapid urbanisation and cropland expansion have reduced connectivity between grasslands, which could have negative consequences for species like *Entomobrya nicoleti* with low dispersal capacity.

### 3.3 Demographic History and Genetic Diversity

**FIGURE 4:**
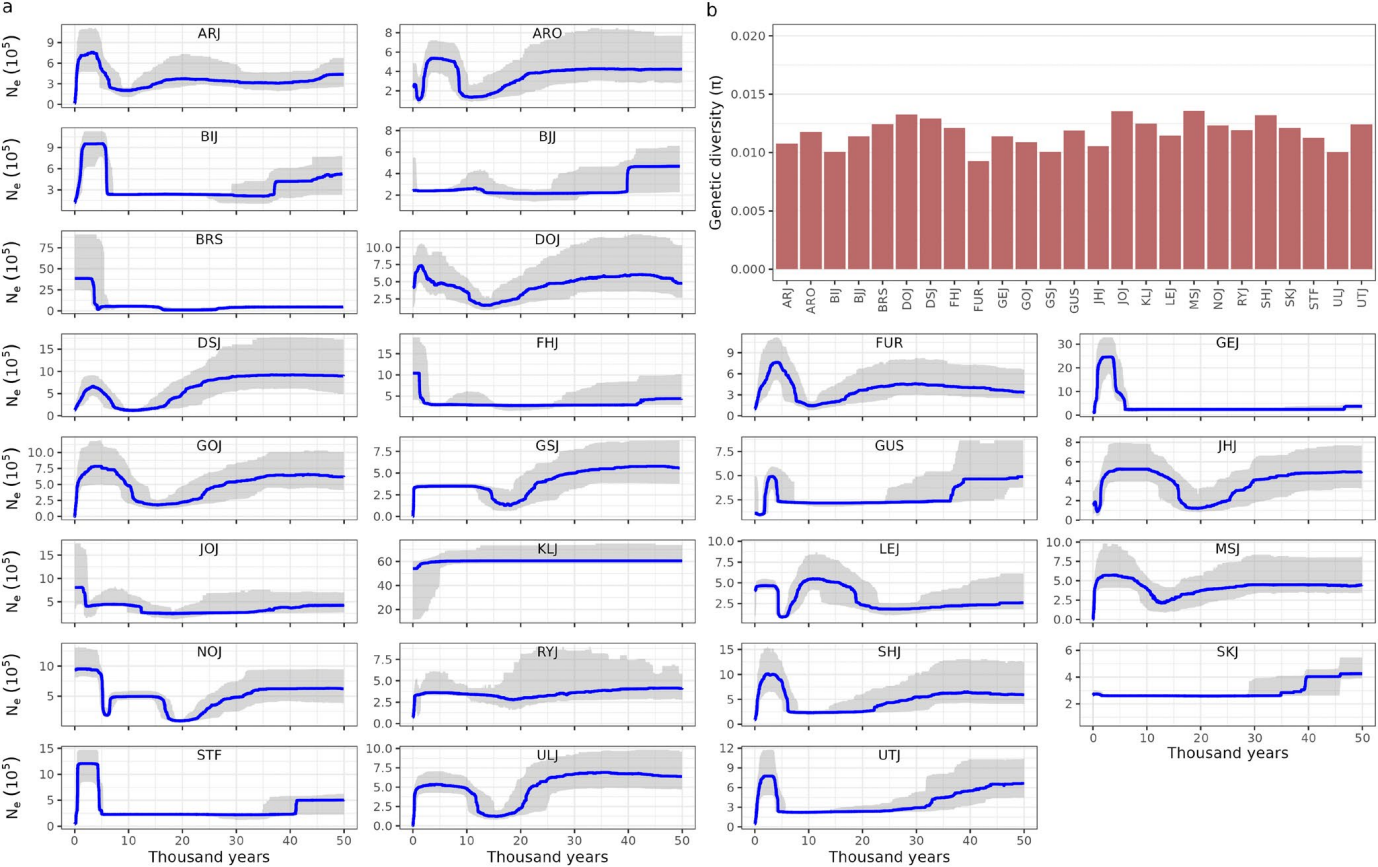
Population demography. Stairway plots (a) showing estimated effective population size (N_e_) for each population from present and 50,000 years back in time. Most populations have experienced drastic decreases in N_e_ in recent times. Note that the y‐axes are scaled with $10^{5}$. Estimates of mean genetic diversity (b), represented as π, are of relatively even magnitude across populations. π was estimated on neutral, mono‐ and bi‐allelic sites.

To investigate the historical changes in effective population size (N_e_) in the 25 sequenced populations we constructed Stairway Plots for each population (Figure 4a). These plots revealed that 18 of 25 populations have experienced increases in population sizes after the glacial retraction (around 10,000 years ago), followed by a drop in size within the last 4000 years which coincides with the intensification of land use. Despite these drops in population size, and the varying demographic histories represented within the populations, genetic diversity is relatively evenly distributed across populations around 1% (π≈0.01, range 0.009–0.013, Figure 4b).

### 3.4 Population Structure

**FIGURE 5:**
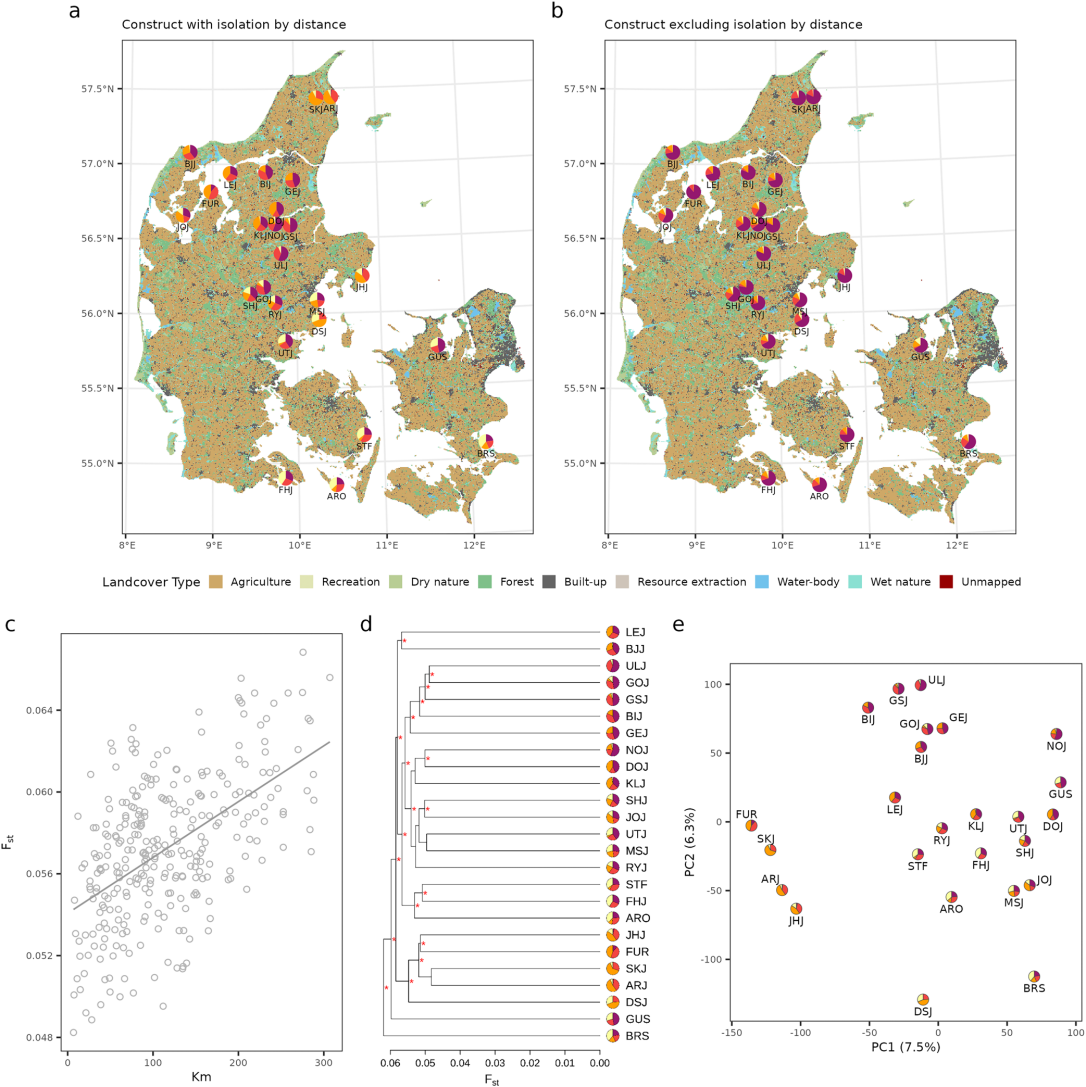
Population structure. Pie charts visualising a conStruct analysis showing clear structure that results from isolation‐by‐distance (a) and no structure when removing the effect of isolation‐by‐ distance (b). The four colours in the pie charts represent each of the four spatial layers (K=4) (c) and deep branches on a UPGMA dendrogram (d) with a red * indicating bootstrap confidence above 70% (1000 replicates). For actual percentages, see Figure S5. A principal component analysis (e) on neutral bi‐allelic variants shows population clustering resembling isolation‐by‐distance.

Across Denmark, populations showed significant isolation‐by‐distance (lm, Radj=0.29, $F\left(1,298\right)=123.6$, p<0.001), while genetic differentiation was relatively low (Figure 5c). Isolation‐by‐distance is clearly visible on a conStruct analysis with isolation‐by‐distance (Figure 5a) while population clustering according to isolation‐by‐distance is shown on the PCA‐plot (Figure 5e). A UPGMA dendrogram based on F_st_ estimations reveals long terminal branches, indicating rapid diversification from a common ancestral population, and limited genetic mixing since (Figure 5d). When investigating the population admixture after removing isolation‐by‐distance, we did not detect additional genetic differentiation (Figure 5b). The diversification between populations is therefore mainly dependent on isolation‐by‐distance.

### 3.5 Gene Flow

The observed genetic structure may reflect a historically stable level of gene flow that ensured connectivity between populations, or a recent decline in gene flow rates among populations, causing a gradual increase in genetic structuring to current levels. To test this, we ran models to estimate demographic parameters for all 300 population pairs. Gene flow rates were estimated to have decreased within the last approximately 10,000 generations (∼ 3300 years), and to be near absent from 5400 to 7200 generations (∼ 1800–2400 years) ago until present (Figure 3). Demographic modelling showed that most population pairs merged into an ancestral population around 9000 years ago (median 9045 years, IQR: 3653:12,917), and that most current effective population sizes were estimated to be 21,856 (diploid, median, IQR: 13,939:27,917), while ancestral effective population sizes were estimated to be much larger, around 1.1 million (diploid, median 1,132,089, IQR: 301,738:2,481,664, Supplemental Figure S4).

The analysis of simulated allele frequencies under a model of decreasing gene flow over time, showed a delay in picking up changes in gene flow rate when looking at recent gene flow (t_2_, present to last 1000 years, Figure S7b). Gene flow during this time (t_2_) could only be detected when t_2_ was higher than 3000 generations (1000 years), despite data being simulated with a gene flow rate of $1\times 10^{-5}$ between 600 and 3000 generations before present. Based on this result we suggest that the gene flow rate estimated on the observed data suffer from a similar ‘delay’ shifting gene flow estimates closer to present times.

Gene flow rates estimated from simulated allele frequencies of both constant gene flow and decreasing gene flow showed that estimated gene flow rates are underestimated compared to the actual simulated gene flow rates, however, the temporal trends of constant and decreasing gene flow were represented well in the analysis despite this (see Figure S7). This suggests that the estimated gene flow of our observed data represent true trends, namely that gene flow rates have been decreasing over the last approximately 4000 years, but also that the actual gene flow rates have been larger than the estimated rates. Moreover, current N_e_ estimates of simulated data are overestimated by approximately a factor 3.7 (input: 11,250, estimates: ∼ 41,998, diploid), suggesting that our estimates from the observed data are overestimated.

## 4 Discussion

It is well documented that landscapes have become increasingly fragmented due to human activities over the last millennia, a phenomenon that has accelerated during the last centuries (Goldewijk et al. 2011; Lohrum et al. 2024; Nielsen et al. 2021; Odgaard and Rasmussen 2000). The area reserved for naturally developing habitats in Denmark is very limited and highly fragmented (Figure 1b). Our sampling suggests that extant populations of the low‐dispersing *Entomobrya nicoleti* are restricted to grasslands as they were not found in conventional agricultural fields, leaving the species with a highly fragmented distribution. The fact that conventional agricultural fields appear as unsuitable habitat for this collembolan species is supported by reports of major negative impacts that tilling has on soil arthropods (Holland 2004; Holland and Reynolds 2003; Thorbek and Bilde 2004) including reductions in collembola abundances of up to 90% (Axelsen et al. 2022). These dramatic negative effects reduce the likelihood that gene flow can take place across conventional agricultural fields. Such dispersal restriction will also limit recolonisation of local populations after potential extinction events (Levins 1969) and further reduce effective population sizes (N_e_) and ultimately standing genetic variation.

Through demographic modelling, we showed that effective population sizes have declined drastically within the last few thousand years in most populations (Figure 4a). Reduction of N_e_ can be a result of populations experiencing restrictions in habitat sizes or habitat suitability, which will lower carrying capacity for species in the habitat (Lynch et al. 1995), and increase isolation of populations. We note that a few populations are not inferred to have been declining in recent years. This may be a result of local habitats that have maintained large and/or in high quality. The increased fragmentation of grasslands in Denmark due to changes in land use within the last few millennia fits well with modelled reductions in N_e_ for most populations investigated (Figures 3 and 4). While other factors such as climate change can have similar effects on the genetic composition of species, this leads us to suggest that reductions in habitat size, quality and connectivity may be an important driver for the general decline in N_e_ for *E. nicoleti*. Most populations seem to have been affected by the last ice age that ended about 10,000 years. This may reflect that Denmark was recolonized when the ice retreated, and that population sizes subsequently expanded until most of them recently declined as discussed above. A direct effect of a decrease in N_e_ is a magnified impact of genetic drift, which according to the Neutral Theory is predicted to accelerate loss of genetic diversity (Kimura 1969). Our estimates of genetic diversity were relatively similar across all populations (π ranging from 0.009 to 0.013, Figure 4b), and comparable to other arthropod species (Leffler et al. 2012). Genetic diversity estimates around 1% are therefore not alarmingly low and is maybe unexpected considering the recent declines in N_e_ estimated for the *E. nicoleti* populations (Figure 4a). An important concern here is that populations having experienced recent declines are expected to keep losing genetic diversity for an extended number of generations, the so‐called ‘drift debt’ (Gargiulo et al. 2024; Gilroy et al. 2017). Our results suggest that measuring current putative neutral genetic diversity may not be sufficient to evaluate whether a population is in the process of losing genetic variants, which could push it into a negative spiral with reinforcing declines in N_e_ and genetic diversity. Populations that experience these processes suffer from decreased evolutionary potential and enhanced risk of extinction (Lynch et al. 1995; Willi et al. 2006).

One of the evolutionary forces that can counteract the potential detrimental effects of small N_e_ is gene flow from neighbouring populations, which can bring new genetic variants or prevent the loss of existing ones. Fastsimcoal2 analyses strongly suggest that gene flow has dramatically decreased among populations of *E. nicoleti*, at least within the last 2000–3000 years (Figure 3). This coincides with intensified land use and agriculture that has led to a highly fragmented landscape in Denmark (Lohrum et al. 2024; Nielsen et al. 2021). We note that the temporal changes in both genetic estimates and in the onset of cropland cover cannot be estimated with exact certainty, due to uncertainty in mutation rate, generation time, calling of singletons, and precision in temporal cropland cover data used.

The current lack of gene flow among *E. nicoleti* populations, which may be driven by habitat fragmentation, can have detrimental consequences in the future (Kardos et al. 2021). First, decreased gene flow may lead to the fixation of deleterious genetic variants (drift load) and thereby reduce fitness. This effect is potentially substantial since most *E. nicoleti* populations used to be much larger and consequently carried a large number of deleterious variants that may be expressed when N_e_ is reduced. At present, relatively high estimated effective population sizes suggest that this may be prevented; however, if population sizes keep decreasing at the pace suggested in this study during the last millennia, this may be problematic. However, our simulations suggest that we overestimate current effective population sizes in the fastsimcoal2 analyses, suggesting that the fixation of deleterious alleles may be a real problem. Second, a lack of gene flow to diminished populations may reduce adaptive genetic diversity, thereby reducing the evolutionary potential of these populations but also preventing beneficial variants that originate locally from spreading to other populations. This may be especially critical for population viability considering the ongoing unprecedented rate of environmental change, which could challenge population persistence. While the lack of gene flow is expected to increase genetic structure among populations, we did not find strong genetic structuring among current populations (Figure 5b,c). Although our fastsimcoal2 results indicate that the current N_e_ is not alarmingly low, it also shows that it was much higher in the past. Since population isolation has occurred relatively recently, it would take more time for its impact on population structure to become evident. The differentiation among populations is relatively small, likely reflecting relatively recent isolation and large population sizes following one large coherent population. Furthermore, the almost equally long terminal branches on the F_st_ cladogram (Figure 5d) suggest that the studied populations have separated relatively quickly since the most recent common ancestor and evolved independently since that. In combination, this supports a recent decline in gene flow coinciding with the intensification of land use.

Small organisms with low dispersal abilities are vulnerable to physical barriers that prevent gene flow. However, our admixture analysis (Figure 5) did not identify any genetic structure further than that caused by isolation by distance. This suggests that gene flow has historically not been prevented by any large barriers, despite some populations being separated by rivers and sea. Ancestrally, it seems that all populations of *E. nicoleti* belonged to a single genetic lineage that has diverged into more or less independently evolving lineages due to recent isolation caused by intensified land use (Figure 5a,c). Since the last ice age, *E. nicoleti* population sizes have expanded into one large continuous population across Denmark. Suitable habitats would have been much better connected at this time, facilitating gene flow on local scales. In large populations of organisms with relatively low dispersal, such as *E. nicoleti*, local drift will be strong enough to overcome homogenisation from gene flow. This results in isolation by distance (Hardy and Vekemans 1999), as also demonstrated here. Turning to the present, we propose that fragmentation due to land use changes in Denmark within the last centuries has resulted in reductions of population sizes and rates of gene flow between populations. The genetic consequences of these reductions are unlikely to have taken full effect yet owing to the drift debt effect (Gargiulo et al. 2024; Gilroy et al. 2017), consistent with the levels of genetic diversity estimated in this study in *E. nicoleti* populations. In the future, *E. nicoleti* could be facing devastating effects of reduced gene flow, which causes a reduction in population sizes (Gomulkiewicz and Holt 1995) and the realisation of genetic load due to inbreeding (Bertorelle et al. 2022; Kardos et al. 2021). This calls for initiatives to establish stable and better‐connected populations for *E. nicoleti* and species with similar life history characteristics to avoid further population size decreases and genetic erosion.

Our study presents novel analyses that substantiate a link between consequences of severe habitat loss and fragmentation to population genetic composition and decline in gene flow of small and relatively abundant species such as collembola. We demonstrate that even species with large population sizes and relatively high levels of genetic diversity can be highly impacted by accelerating habitat fragmentation if their dispersal capacity is low. Although we detect severe declines in N_e_ and gene flow, current levels of genetic diversity are not alarmingly low. However, these demographic processes predict future loss of genetic diversity and genetic erosion, and the study highlights that negative consequences have yet to manifest and are likely to also be extremely detrimental for common species in fragmented landscapes. In the light of conservation genetics, the results of this study call for management decisions to be based on in‐depth genomic studies of genetic diversity and structure, in addition to demographic trajectories.

## Author Contributions

Tammy Ai Tian Ho: data collection, manuscript drafting. Jeppe Bayer Pedersen: data analyses, bioinformatics, manuscript drafting. Anne Aagaard: data analyses, figures, bioinformatics, manuscript drafting. Mads F. Schou: data analyses, manuscript drafting. Jesper Bechsgaard: design of study, data analyses, bioinformatics, manuscript drafting. Derek Corcoran: landscape and land use change analyses, manuscript drafting. Tove Hedegaard Jørgensen: data analyses, manuscript drafting. Signe Normand: landscape and land use change analyses, manuscript drafting. Trine Bilde: design, coordination and planning of study, manuscript writing.

## Conflicts of Interest

The authors declare no conflicts of interest.

## Supporting information


Data S1.

## Data Availability

The assembly and annotation of the *Entomobrya nicoleti* genome (FASTA format), and re‐sequencing data (FASTQ format) are uploaded to NCBI under BioProject ID: PRJNA1132793 http://www.ncbi.nlm.nih.gov/bioproject/1132793. All code used for this study is available at: https://github.com/EcologicalGenetics/gene_flow_disruption_and_population_fragmentation_in_a_soil_arthropod_in_fragmented_habitats.
