# Supplementary material for: Gene Flow Disruption and Population Declines in a Soil Arthropod in Fragmented Habitats
Source: Mol Ecol. 2025 Jun 11;34(23):e17820. doi: 10.1111/mec.17820 (PMC12684311; doi:10.1111/mec.17820)
Supplement: Supplementary file 1 — Data S1. [file MEC-34-e17820-s001.pdf]

MOLECULAR ECOLOGY

Supplemental information for:

Gene Flow Disruption and Population Declines in a Soil Arthropod in Fragmented Habitats

Tammy Ai Tian Ho, Jeppe Bayer Pedersen, Anne Aagaard, Mads F. Schou, Jesper Bechsgaard, Derek Corcoran, Tove Hedegaard Jørgensen, Signe Normand, Trine Bilde

Table of Contents:

Figure S1	1
Figure S2	1
Figure S3	2
Figure S4	3
Figure S5	4
Figure S6	5
Figure S7	6
Table S1	8
Table S2	9

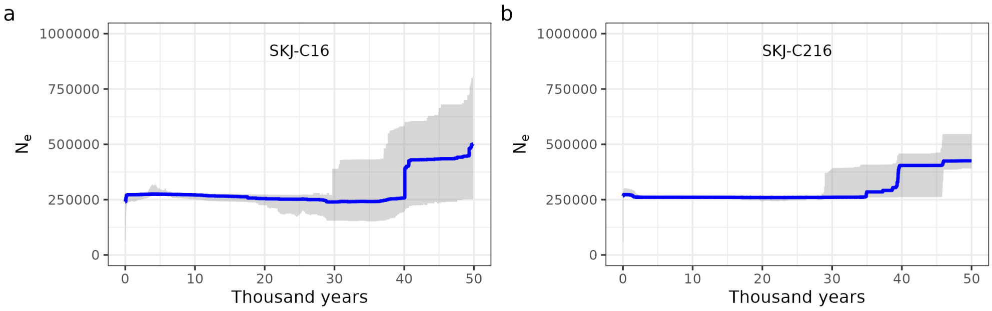

Figure S1: Stairway Plots for two samples from SKJ. Stairway Plots comparing effective population size (N_e) trajectories of a population where 50 individuals were collected twice, and each of these samples were sequenced and analysed for comparison. The two trajectories show highly similar topology and population size estimations.

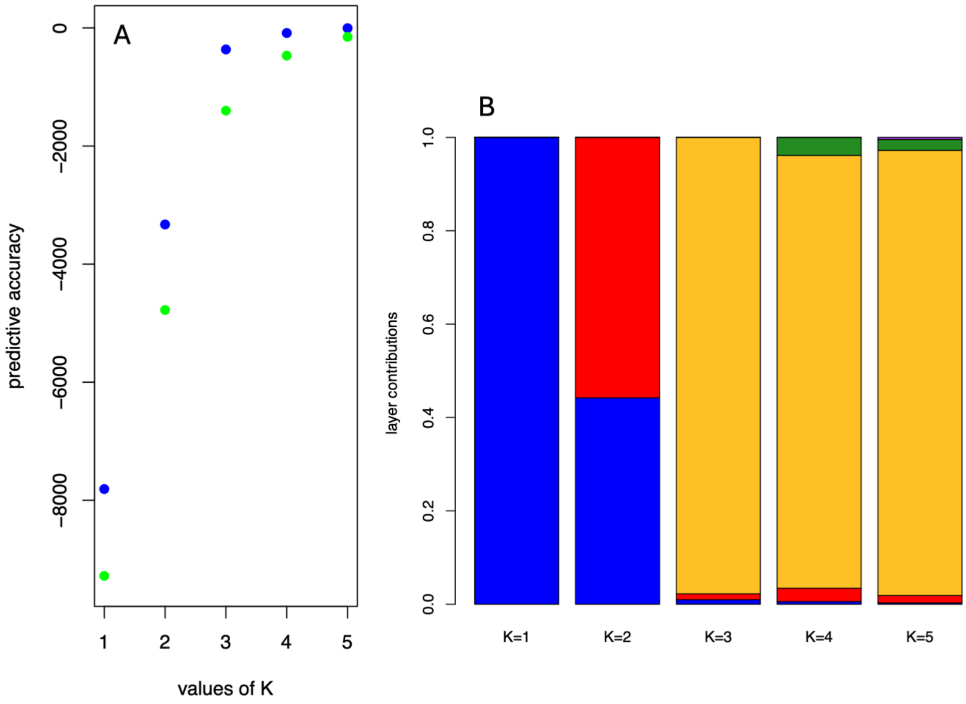

Figure S2: conStruct analysis cross validation. (A) Change in predictive accuracy of conStruct models with (blue) and without (green) isolation by distance (IBD), and with 1 to 5 spatial layers (K's) using cross-validation in 16 replicate models. Confidence intervals (95%) are too small to be visible. Predictive accuracy was strongest for models that included IBD and we therefore inspected layer contributions for these models (B). Based on the minor increase in predictive accuracy from $K = 4$ to $K = 5$, and the limited change in layer contributions, we find that the model with $K = 4$ best reflects the data and therefore present more detailed population level layer contributions of this model in the main manuscript.

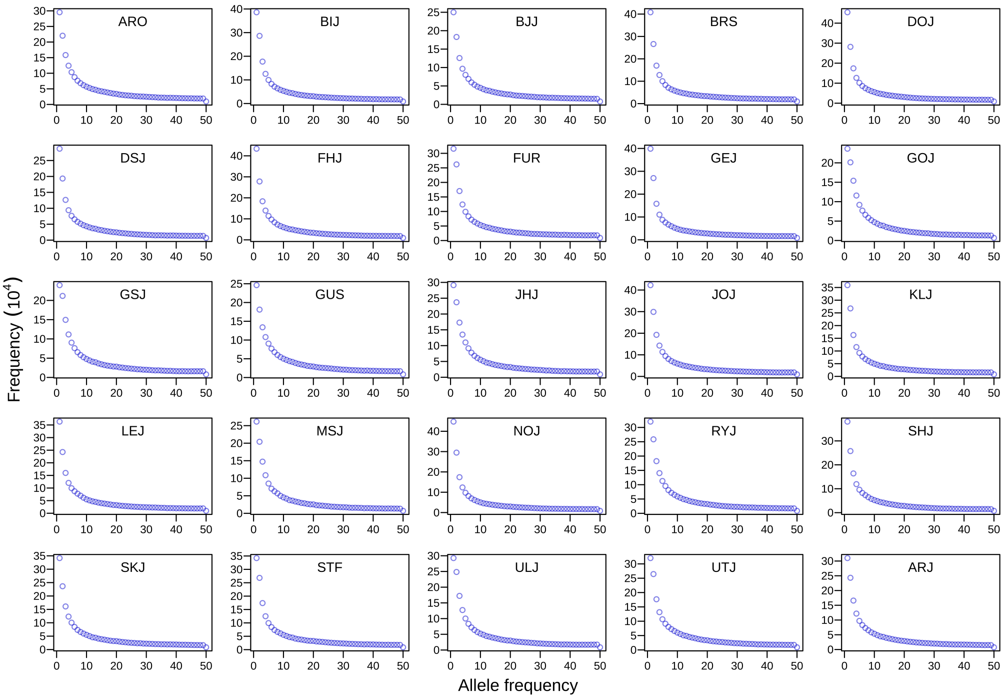

Figure S3: Site frequency spectra. Site frequency spectra for the 25 analysed populations of *Entomobrya nicoleti*.

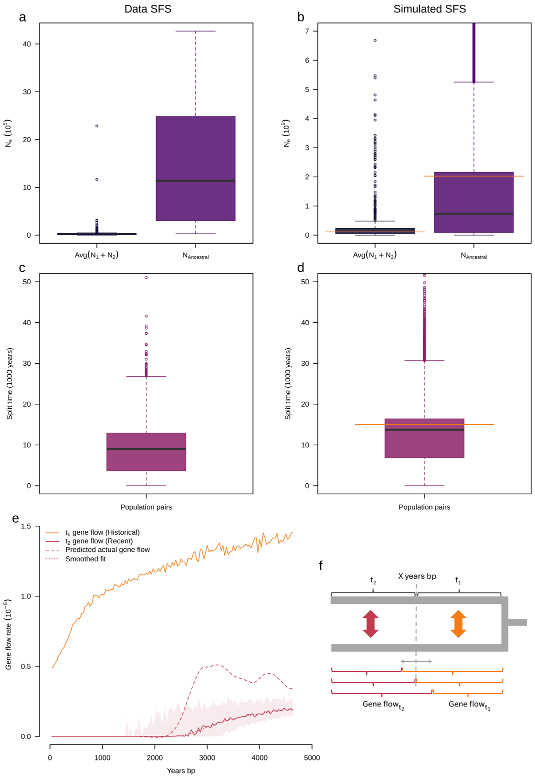

Figure S4: fastsimcoal2 model estimates. Visualisation of fastsimcoal2 estimates related to the model in Figure 1. Bar-plots showing effective population size estimates for current and ancestral populations from (a) observed site frequencies (diploid, median: current = 21,856, IQR: 13,939:27,917, ancestral = 1,132,089, IQR: 301,738:2,481,664) and (b) simulated site frequencies (diploid, median: current = 20,855, IQR: 4,813:22,561, ancestral = 73,395, IQR: 9,066:215,521). The horizontal orange lines in (b) indicate the effective population sizes that the simulation was based on (current N_e = 11,250, ancestral N_e = 202,500, diploid). As can be seen from (b), current effective population sizes seem overestimated, and we assume this is true for the observed data as well. Estimates of when the ancestral population split into two populations are shown as bar-plots of (c) observed data (median = 9,045 years (27,135 generations), IQR: 3,653:12,917), and (d) simulated data (median = 13,748 years (41,241 generations), IQR: 6,882:16,397). The solid orange line (d) shows input split time for the simulation (15,000 years (45,000 generations)). Gene flow estimates from observed data are shown in (e) as two curves (orange and red solid lines, shaded area indicates 95% CI), estimated for all population pairs in steps of 33.3 years (100 generations). The fastsimcoal2 model employed (f) estimates two gene flow rates per X years, a gene flow rate integrated over the time from population split to X (t_1, orange), and a gene flow rate integrated over the time from X to present (t_2, red). We then estimate these two rates in moving X in steps of 33.3 years (100 generations) from the present to ~ 4,666 years before present (bp) (14,000 generations). Population pairs with split time less than 14,000 generations were discarded from further gene flow rate analyses. Since the gene flow rate estimates shown in (e) are integrated over varying time intervals, we estimated the actual gene flow rate for the t_2 gene flow estimates, by fitting a smoothed line (dotted red line), and calculating the actual gene flow rate for step along the x-axis (dashed red line). The actual values were calculated using equation 2 in section 2.3.7 in the manuscript.

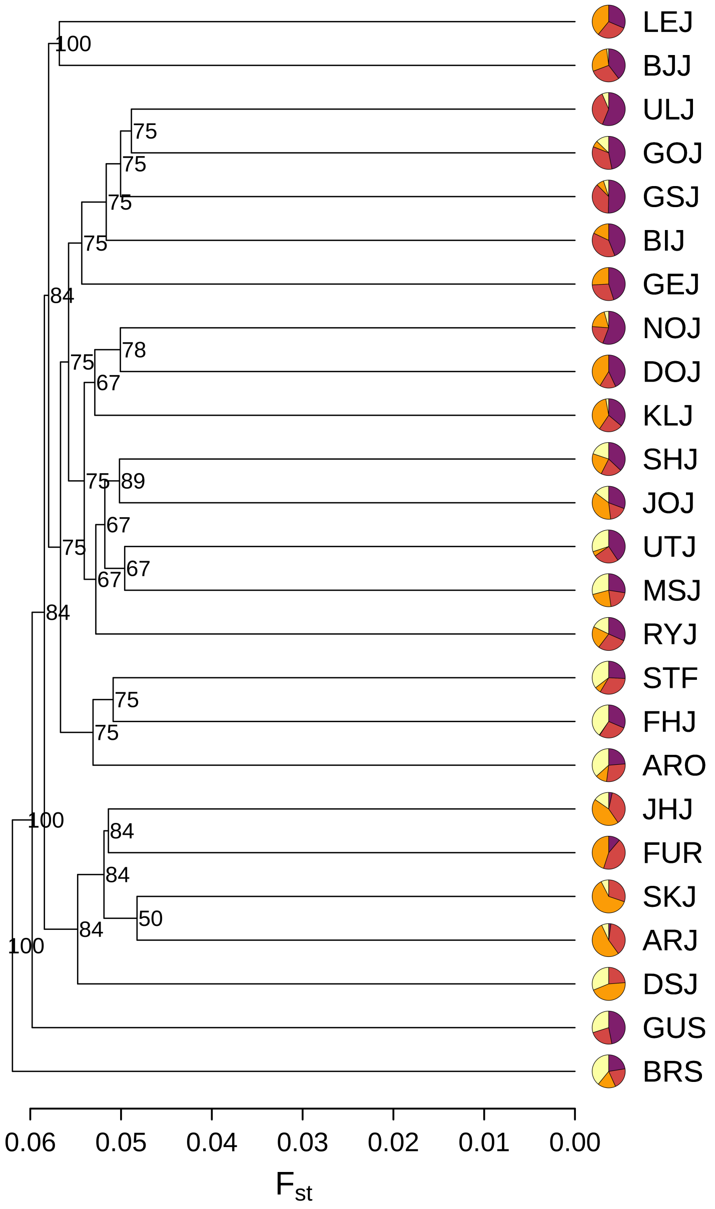

Figure S5: UPGMA Dendrogram with bootstrap confidence as percentages. UPGMA dendrogram based on F_{st} values estimated from neutral variable sites with sufficient coverage in all populations. As opposed to the dendrogram in the manuscript, bootstrap confidence has been added as actual percentages (1,000 replicates). Pie-charts show conStruct analysis including isolation by distance.

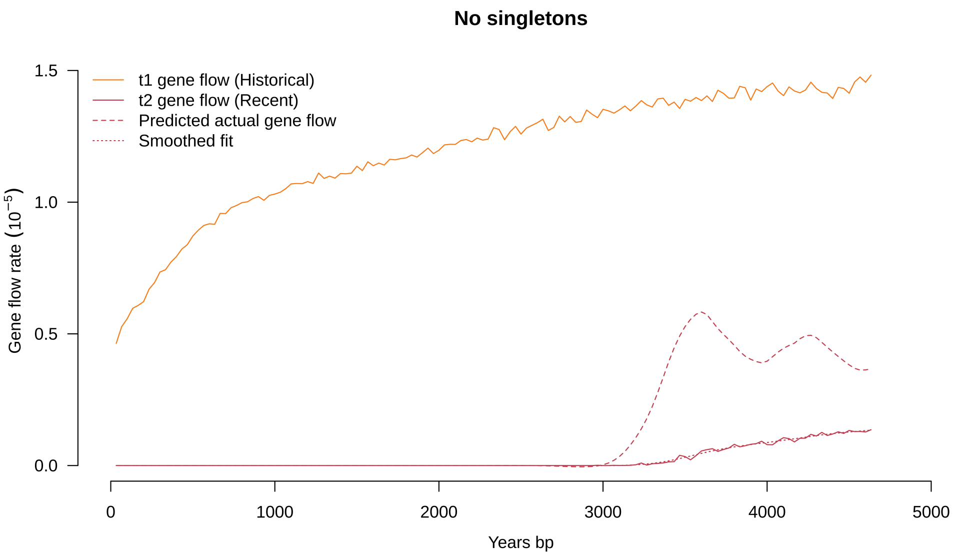

Figure S6: Gene flow analysis without taking singletons into account. The analysis explained in Figure S4 was replicated using the option `--nosingleton` in fastsimcoal2. For a more detailed explanation, see Figure S4 and section 2.3.7 in the manuscript. The analysis showed similar trajectories to the analysis including singletons (Figure S4e), while the timing is shifted $\sim$ 500 years back in time. Singletons do not seem to affect the analysis much.

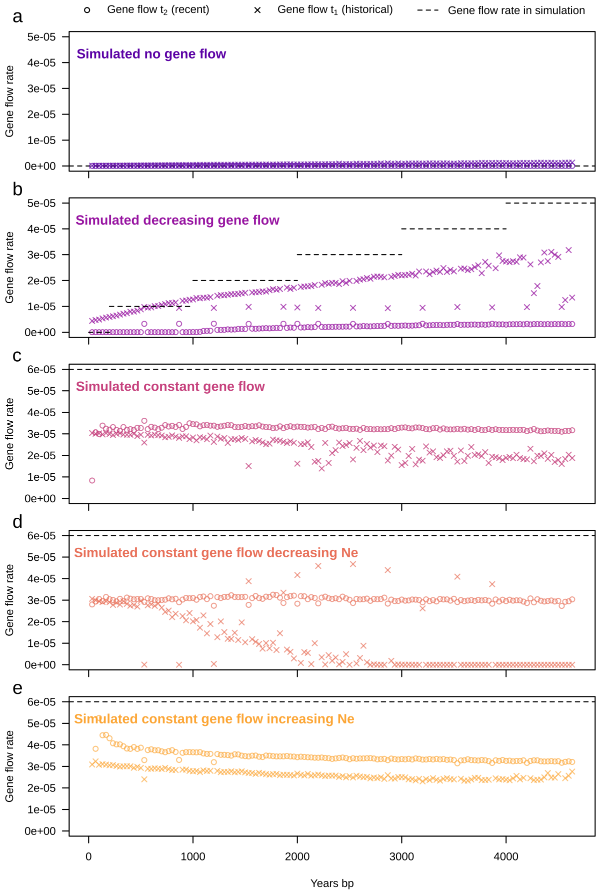

Figure S7: fastsimcoal2 gene flow rate estimates from simulated site frequency spectra. Graphical representation of gene flow estimates based on fastsimcoal2 analysis of simulated site frequency spectra (SFS). fastsimcoal2 was used to generate 100 replicates of SFS for 50 diploid individuals per population under five different evolutionary scenarios: no gene flow (a), decreasing gene flow (b), constant gene flow (c), constant gene flow with decreasing N_e (d), and constant gene flow with increasing N_e (e). The five scenarios were all based on two populations both with a current size of 22,500 (11,250 diploid) that split up 45,000 generations ago, an ancestral population size of 405,000 (202,500 diploid), and with six chromosomes of 15,000,000 bases each, a recombination rate of 1×10^{-8}, and a mutation rate of 1×10^{-8}. The individual parameters were: (a) gene flow of 0, (b) decreasing symmetrical gene flow over the last 12,000 generations (gene flow of 6×10^{-5} from population split until 12,000 generations ago, of 5×10^{-5} from 12,000 to 9,000 generations ago, of 4×10^{-5} from 9,000 to 6,000 generations ago, of 3×10^{-5} from 6,000 to 3,000 generations ago, of 2×10^{-5} from 3,000 to 600 generations ago, of 1×10^{-5} and of 0 between 600 generations ago until present), (c) constant symmetrical gene flow of 6×10^{-5}, (d) constant symmetrical gene flow of 6×10^{-5} and exponentially declining population size with a rate of 0.00005 per generation since the split of the two populations (current size being 1,000 diploid individuals), and (e) constant symmetrical gene flow of 6×10^{-5} and exponentially increasing population size with a rate of 0.00005 per generation since the split of the two populations (current size being 500,000 diploid individuals).

**Grasslands**			
Location name	Location code	Sampling effort (N times)	Individuals collected (N)
Årsted	ARJ	4	50
Ærø	ARO	4	50
Bislev	BIJ	4	50
Bjerre	BJJ	4	50
Brønsvig	BRS	4	50
Døstrup	DOJ	3	50
Dyngby Strand	DSJ	5	50
Fårup	FAJ	3	39
Fem Høje	FHJ	5	50
Fur	FUR	4	50
Gerding	GEJ	4	50
Gødvad	GOJ	4	50
Glenstrup Sø	GSJ	3	50
Gurede	GUS	4	50
Hæsum	HAJ	4	1
Havelse	HAS	5	2
Hedeland	HES	4	2
Hørhaven	HHJ	8	50
Hylkedam	HYF	5	8
Jelling	JEJ	4	50
Jernhatten	JHJ	7	50
Jegindø	JOJ	4	50
Jyllinge	JYS	5	22
Klejtrup	KLJ	4	50
Kalø	KOJ	6	50
Kongskilde	KOS	5	35
Kørup	KRJ	4	0
Korsør	KRS	4	37
Lendrup	LEJ	4	50
Lille Vildmose	LVJ	4	50
Mogenstrup	MOS	5	16
Moesgaard Strand	MSJ	9	50
Mullerup Strand	MSS	4	47
Myrdeskov	MYS	5	13
Nørre Onsild	NOJ	4	50
Nysted	NYS	3	0
Orø	ORO	4	46
Rydal	RDJ	4	12
Regnemark	RES	4	44
Røde Mølle	RMJ	2	0
Ry	RYJ	7	50
Skindbjerg	SBJ	3	50
Skjærskovhede	SHJ	3	50
Skærum	SKJ	4	50
Skovly	SKS	4	27
Stubbegård Sø	SSJ	2	28
Stokkebæk	STF	6	50
Stenhøj	STJ	3	25

Location name	Location code	Sampling effort (N times)	Individuals collected (N)
Suldrup	SUJ	4	22
Tolstrup	TOJ	4	27
Torrild	TRJ	5	33
Ulstrup	ULJ	4	50
Uth	UTJ	4	50
Vandel	VAJ	3	50
Vigersdal	VIS	5	2
**Conventional agricultural fields**			
Bredvadmølle	K_BMJ	2	0
Dalby	K_DAS	2	0
Flemming	K_FLJ	2	0
Fladstrup	K_FSJ	2	0
Hvam	K_HVJ	2	0
Hylstrup	K_HYS	2	0
Jerslev	K_JES	2	0
Knebel	K_KNJ	2	0
Lerchenborg	K_LES	2	0
Rødkilde	K_ROF	1	0
Sterup	K_STJ	1	0
Tørring	K_TOJ	2	0
Yding	K_YDJ	2	0

Table S1: Sampling effort. Sampling sites and sampling effort for grasslands, conservation agriculture and conventional agriculture. This table was updated until the start of data analysis in March 2024. We aimed at sampling 50 individuals per sampling site. Samples where the target was reached were sent for sequencing. During analysis, samples where less than 50% of the genome was covered (grey background, $n = 6$) after filtering showed elevated number of variant sites and proportions of non-synonymous mutations and were therefore removed from all subsequent analyses, so the full dataset constitute 25 grassland samples (white background).

Genome Assembly

Assembly level	Chromosome level	All contigs
Specimen	*Entomobrya nicoleti*	*Entomobrya nicoleti*
Isolate	Pooled 20 individuals (males + females)	Pooled 20 individuals (males + females)
Sequence coverage	69	69
Genome size (Mb)	254.1	372.3
Gaps	0.604%	1.248%
Number of contigs	3,187	6,460
Contig N50 (Kb)	150	115
Number of chromosomes	7	8
Number of protein-coding genes	31,573	40,432
Mean exon length (Bp)	367	338
Total exon length (Mp)	40.2 (15.83%)	53.1 (14.27%)
Number of exons	109,588	157,329
Mean intron length (Bp)	409	416
Total intron length (Mb)	31.9 (12.56%)	48.6 (13.05%)
Number of introns	78,015	116,901
Number of exons per gene	3.5	3.9
GC content	34.27%	34.42%
Repeat content	20.77%	21.56%
Unclassified repeat content	14.62%	15.13%
BUSCO* genome score	C:50.9%[S:48.9%,D:2.0%], F:2.4%,M:46.7%,n:1013	C:93.9%[S:90.5%,D:3.4%], F:1.8%,M:4.3%,n:1013

Table S2: The inclusion of all contigs added 3,273 contigs to the assembly, these were assembled into 3,081 scaffolds. To ease future downstream analyses these scaffolds were concatenated into a single superscaffold with 1,000 N's inserted between each scaffold. This has inflated the percentage of gaps from 0.424% to the indicated 1.248%. The original structure is preserved in an accessory BED file. *BUSCO scores based on the arthropoda_odb10 BUSCO set using v5.5.0 C=complete [S=single copy, D=duplicated], F=fragmented, M=missing, n=number of orthologues in comparison.
